# Spatial genetic structure of European wild boar, with inferences on late-Pleistocene and Holocene demographic history

**DOI:** 10.1038/s41437-022-00587-1

**Published:** 2023-01-13

**Authors:** Joost F. de Jong, Laura Iacolina, Herbert H. T. Prins, Pim van Hooft, Richard P. M. A. Crooijmans, Sip E. van Wieren, Joaquin Vicente Baños, Eric Baubet, Seán Cahill, Eduardo Ferreira, Carlos Fonseca, Peter M. Glazov, Ida Jelenko Turinek, Victor M. Lizana Martín, András Náhlik, Boštjan Pokorny, Tomasz Podgórski, Nikica Šprem, Rauno Veeroja, Ronald C. Ydenberg, Hendrik-Jan Megens

**Affiliations:** 1 Wildlife Ecology & Conservation Group, Wageningen University, Droevendaalsesteeg 3A, 6708 PB Wageningen, The Netherlands; 2 Department of Chemistry and Bioscience, Aalborg University, Frederik Bajers Vej 7H, 9220 Aalborg, Denmark; 3 Faculty of Mathematics, Natural Sciences and Information Technologies, University of Primorska, Glagoljaška 8, SI-6000 Koper, Slovenia; 4 Animal Sciences Group, Wageningen University, De Elst 1, 6708 WD Wageningen, The Netherlands; 5 Animal Breeding and Genomics Group, Wageningen University, Droevendaalsesteeg 1, 6708 PD Wageningen, The Netherlands; 6 SaBio, Instituto de Investigación en Recursos Cinegéticos -IREC (CSIC-UCLM-JCCM), Ronda de Toledo 12, 13071 Ciudad Real, Spain; 7 Office Français pour la Biodiversité, Monfort, 01330 Birieux France; 8 Estació Biològica de Can Balasc, Consorci del Parc Natural de la Serra de Collserola, Ctra. de l’Església 92, 08017 Barcelona, Spain; 9 Department of Biology & CESAM - Centre for Environmental and Marine Studies, University of Aveiro, Portugal e de Aveiro, Portugal; 10 Institute of Geography, Russian Academy of Sciences, Staromonetny per. 29, Moscow, 119017 Russia; 11 Ministry of Environment and Spatial Planning, Dunajska 48, 1000 Ljubljana, Slovenia; 12 Servicio de Análisis, Investigación y Gestión de Animales Silvestres (SAIGAS), Facultad de Veterinaria, Universidad Cardenal Herrera-CEU, CEU Universities, C/Tirant lo Blanc 7, 46115, Alfara del Patriarca, Valencia, Spain; 13 Wildlife Ecology & Health group (WE&H), Universitat Autònoma de Barcelona (UAB), Edifici V, Travessera del Turons, 08193 Bellaterra Barcelona, Spain; 14 University of Sopron, Bajcsy-Zsilinszky u. 4., H-9400 Sopron, Hungary; 15 Sapientia Hungarian University of Transylvania, 400112 Cluj-Napoca, str. Matei Corvin nr. 4, Romania; 16 Faculty of Environmental Protection, Trg mladosti 7, 3320 Velenje, Slovenia; 17 Slovenian Forestry Institute, Večna pot 2, 1000 Ljubljana, Slovenia; 18 Mammal Research Institute, Polish Academy of Sciences, Stoczek 1, 17-230 Białowieża, Poland; 19 Department of Game Management and Wildlife Biology, Faculty of Forestry and Wood Sciences, Czech University of Life Sciences, Kamýcká 129, 165 00 Praha 6, Czech Republic; 20 Department of Fisheries, Apiculture, Wildlife Management and Special Zoology, Faculty of Agriculture, University of Zagreb, Svetošimunska cesta 25, 10000 Zagreb, Croatia; 21 Estonian Environment Agency, Rõõmu tee 6, 50705 Tartu, Estonia; 22 Centre of Wildlife Ecology, Simon Fraser University, Burnaby, BC V5A 1S6 Canada

**Keywords:** Genomics, Population dynamics, Genetic variation, Evolutionary genetics

## Abstract

European wildlife has been subjected to intensifying levels of anthropogenic impact throughout the Holocene, yet the main genetic partitioning of many species is thought to still reflect the late-Pleistocene glacial refugia. We analyzed 26,342 nuclear SNPs of 464 wild boar (*Sus scrofa*) across the European continent to infer demographic history and reassess the genetic consequences of natural and anthropogenic forces. We found that population fragmentation, inbreeding and recent hybridization with domestic pigs have caused the spatial genetic structure to be heterogeneous at the local scale. Underlying local anthropogenic signatures, we found a deep genetic structure in the form of an arch-shaped cline extending from the Dinaric Alps, via Southeastern Europe and the Baltic states, to Western Europe and, finally, to the genetically diverged Iberian peninsula. These findings indicate that, despite considerable anthropogenic influence, the deeper, natural continental structure is still intact. Regarding the glacial refugia, our findings show a weaker signal than generally assumed, but are nevertheless suggestive of two main recolonization routes, with important roles for Southern France and the Balkans. Our results highlight the importance of applying genomic resources and framing genetic results within a species’ demographic history and geographic distribution for a better understanding of the complex mixture of underlying processes.

## Introduction

In human-dominated landscapes, the genetic variation of wildlife is shaped not only by natural demographic forces, but also by anthropogenic factors. In Europe, the Last Glacial Maximum (LGM), when ice sheets reached their most recent maximum extent ~27k-19k years ago (Clark et al. 2009), is considered a major demographic force, as it restricted most wildlife populations to southern refugia for several thousand years and left genetic signatures that are still detectable today (Hewitt 1999, 2004). Although shorter, a similar force was asserted by humans during the last few centuries, as overexploitation and eradication reduced the ranges and population sizes of wildlife across Europe (Apollonio et al. 2010), whereas changes in land use and increased presence of infrastructures and barriers to animal movement led to fragmentation and loss of connectivity (Apollonio et al. 2010; Deinet et al. 2013; Koemle et al. 2018). On top of this, another anthropogenic impact was asserted via translocation and hybridization events with domestic relatives or introduced (sub)species, (e.g., *Cervus* spp. and *Capreolus* spp.; Putman et al. 2011; Iacolina et al. 2019; De Jong et al. 2020).

One of the European mammals strongly affected by humans is the wild boar (*Sus scrofa*). Although currently widespread, populations were decimated or eradicated until World War II across large part of Europe and later supplemented with individuals from other regions (Apollonio et al. 2010). Additionally, the species has experienced genetic introgression from domestic pigs (*S. s. domesticus*) in many areas of its range, although the degree of genetic ‘pollution’ varies greatly among locations (Goedbloed et al. 2013a; Iacolina et al. 2018, 2019). Genetic studies have shown that, in the absence of barriers to the species, the observed hybridization patterns and genetic discontinuities are likely to be caused by translocation events (Vernesi et al. 2003) or local extinctions (Ferreira et al. 2009; Nikolov et al. 2009; Goedbloed et al. 2013b). However, these alterations of genetic variation were found to be minor compared to signatures caused by the LGM (Scandura et al. 2008, 2011a).

The demographic history inferred from whole genome sequences shows that wild boar populations underwent a marked decline during the Late Pleistocene, reaching the lowest levels around the LGM (Groenen et al. 2012). In line with this, mismatch distributions of mitochondrial DNA (mtDNA) sequences of European wild boar show signs of demographic expansion expected to have occurred when climate became more favorable after the LGM (Scandura et al. 2008; Alexandri et al. 2012), although strong demographic fluctuations were not detected in every country across Europe (Kusza et al. 2014). Additionally, mtDNA haplotype diversity generally decreases with latitude (Vilaça et al. 2014), with higher levels in southern Italy and lower in northwestern Europe. This pattern is indicative of the leading-edge expansion model, in which most of the recolonization is undertaken by descendants of the northernmost populations of refugia (Hewitt 1999; Nykänen et al. 2019). Moreover, each putative refugium has its own unique set of haplotypes, whilst most of the haplotypes observed in northern regions are shared with one or several refugia (Scandura et al. 2008; Alves et al. 2010; Goedbloed et al. 2013b; Vilaça et al. 2014; Veličković et al. 2015). Lastly, eastern European wild boar, like pigs, have 2n = 38 chromosomes, whereas due to Robertsonian fusion of chromosomes 15 and 17, western wild boar typically have 2n = 36 (Rejduch et al. 2003)—suggesting the presence of at least two different European clades. Nevertheless, it remains unclear what exactly the contribution of each refugium was to the recolonization of northern Europe and where and when wild boar populations from different refugia met to form hybrid zones.

Although the LGM has been recognized to have left the strongest signature on wild boar genetic composition (e.g., Scandura et al. 2008, 2011a), human manipulation throughout the Holocene and in particular the last two or three centuries have also contributed to shaping the species’ genetic structure (Scandura et al. 2011b; Goedbloed et al. 2013b). While artificial infrastructures appear to have limited influence on wild boar dispersal and, consequently, on its genetic structure (Frantz et al. 2012; Mihalik et al. 2018), urban environments are becoming increasingly used by this species (Cahill et al. 2012; Stillfried et al. 2017). However, the main anthropogenic drivers affecting genetic diversity were identified as hunting, translocations and reintroductions (Vernesi et al. 2003; Scandura et al. 2011a), together with farming practices that led to domestic pig x wild boar hybridization (Goedbloed et al. 2013a; Iacolina et al. 2018). Studies based on both microsatellites (Vernesi et al. 2003; Scandura et al. 2008, 2011a; Ferreira et al. 2009) and SNPs (Goedbloed et al. 2013b; Iacolina et al. 2016) showed a connection between human-mediated wild boar movement and the observed genetic structure at local scale.

We analyzed genome-wide SNP data of European wild boar to (a) assess to what extent European wild boar populations are subject to pig hybridization, translocation and inbreeding, and to (b) delineate the continental wide spatial genetic structure of wild boar. In so doing, we ultimately aimed to infer (i) to what extent anthropogenic influences have altered the natural spatial genetic structure, and (ii) how and to what extent the spatial genetic structure still holds signatures of the LGM. Given the historic and contemporary intense human influence on European wild boar, we predicted to detect inbreeding, hybridization and translocation events in numerous wild boar populations. Depending on the intensity of these anthropogenic influences, we expected alteration of the spatial continental-wide genetic structure through weakening or erasing of natural signatures such as Isolation by Distance, and—in case of geographic barriers – Isolation by Resistance patterns. For the LGM specifically, we expected to find genetic signatures reminiscent of refugia, recolonization areas and (a) suture zone(s). An overview of our hypotheses is presented in Table S1.

## Materials and methods

### Sample collection and genotyping

To obtain a good coverage of the European continent, we combined publicly available SNP data on European mainland wild boar (Iacolina et al. 2016) with newly collected samples from previously underrepresented regions, and with genetically distinct populations as reference (Table S2). All samples were collected within the frameworks of national game management and population control programs according to national laws.

For detection of pig introgression, we incorporated 140 pig samples, which were collected within the framework of the PigBioDiv project (see Megens et al. 2008). These pig samples came from four standardized domestic breeds (Large White, Landrace, Angler Sattle and Pietrain), three Spanish (Negro Iberico, Manchado and Retinto) and three Italian indigenous breeds (Calabrese, Casertana and Cinta Senese).

We isolated DNA following the Gentra Puregene Blood kit protocol (Qiagen, Venlo, the Netherlands). Samples were genotyped using the PorcineSNP60 DNA Analysis Kit beadchip (https://emea.illumina.com/products/by-type/microarray-kits/porcine-snp60.html). After excluding duplicates and individuals with call rates below 0.95, we retained 464 animals from 23 different countries in mainland Europe (Table S2). Additionally, we had 15 wild boar from Israel and the Greek island of Samos (Near East lineage) and 33 samples from the East-Asian lineage (Korea, Japan, eastern Russia and China) for comparison. Since two versions of the PorcineSNP60 beadchip are available (v1 and v2), and some of the previously published data were genotyped with version v1, we verified that genotypes of both beadchip versions aligned and were hence compatible (see Fig. S1 for additional information) by means of a Principal Coordinate Analysis (PCoA) on pairwise genetic distances in Adegenet 2.1.0 (Jombart and Ahmed 2011).

### Quality control

We focused our analyses on the 26,342 autosomal SNPs that occurred on both PorcineSNP60 beadchip v1 and v2, had call rates above 0.95 and minor allele frequency (MAF) above 0.025. Finally, to adhere to the assumption of neutrality, for all analyses except runs of homozygosity (ROH), we excluded intragenic SNPs (remaining: 15,296 SNPs).

We used PLINK (Purcell et al. 2007; Chang et al. 2015) to remove SNPs with strong dependency (LD) (function *indep-pairwise*, window of 50 SNPs, step size 5). Two filtering procedures were used, a more relaxed r^2^ < 0.5 for ROH analyses (21,261 intra- and intergenic SNPs remaining) and a more stringent r^2^ < 0.2 (9761 intergenic SNPs remaining) to investigate genetic variation and structure. Additionally, we used the same software to calculate, separately for each sampling location, relatedness among individuals as an identity-by-descent score using subsets of SNPs with MAF > 0.10. For subsequent analyses, we retained only non-related individuals (identity-by-descent score <0.183). This filtering procedure led to a dataset of 330 wild boar from 56 sampling locations across mainland Europe (minimum, median and maximum sample size per location: 1, 5 and 26, respectively).

### Hybridization and inbreeding

To estimate the intensity of introgression from pigs, we used the hybrid detection technique called ‘PCoA projection’ (see McVean 2009), where observed wild boar genotypes are projected on a polar axis that opposes pig genotypes and simulated ‘pure’ wild boar genotypes. Genotypes of pure wild boar were simulated through application of the R 3.4.2 (R Core Team 2018) base function rbinom (‘n’ = 20 simulated pure wild boar, ‘size’ = 2 alleles, ‘prob’ = a vector with, for each locus, the MAF observed at population level, which, unless the whole population is hybridized, represents a wild boar signature). Because of the existence of genetic structure among populations (see Results), MAFs were estimated separately per genetic cluster (four clusters: Iberia, southern France, Italy, and rest of Europe) (Fig. S2). To subsequently estimate genetic distances among pigs, the simulated pure wild boar and the observed wild boar samples, we calculated Hamming pairwise genetic distances using the R package poppr 2.8.0 (Kamvar et al. 2014). Subsequently, we did a PCoA analysis using the R package ape 3.0 (Paradis et al. 2004). In accordance with McVean (2009), the projected ordination positions of the observed wild boar samples towards the pig source population were then taken as a proxy for percentage of pig ancestry. Furthermore, following Goedbloed et al. (2013a), we further explored pig introgression by examining the distribution of alleles that are uncommon in European wild boar (MAF < 0.025), but abundant in pigs (MAF > 0.225). We studied whether these putative pig alleles had a higher frequency in the hybrids detected by PCoA projection. Additionally, we examined the distribution of these alleles along the genome, thereby verifying clustering of pig alleles in certain regions of the genome (Fig. S3), which is indicative of introgressed haplotypes (Goedbloed et al. 2013a).

To study the level of inbreeding, we used the fraction of the genome containing ROH segments (F_ROH_). F_ROH_ can be reliably estimated with 10,000 SNPs or more, if targeted at large ROH segments (Kardos et al. 2018). We used the 21,287 SNPs with relaxed LD filtering and focused on >5 Mb regions (common ancestor maximum 10 generations ago; Kardos et al. 2018) that had a minimum of 50 SNPs with a mean density of at least 1 SNP per 150 kb and a maximum inter-SNP distance of 500 kb (Fig. S4). We detected ROHs using the *homozyg* function in PLINK and criteria of 70 consecutive SNPs, with no heterozygotes allowed (Howrigan et al. 2011; Ferenčaković et al. 2013). We excluded wild boar samples for which F_ROH>5Mb_ was larger than 0.125 as that is the expected level of autozygosity for offspring of 2^nd^ order relatives.

### Spatial genetic structure

We examined spatial genetic structure through PCoA and ADMIXTURE 1.3.0 (Alexander et al. 2009), the latter plotted against a European map of historic forest cover (year: 1850, Kaplan et al. 2009). To prevent inbreeding and hybridization from confounding the genetic structure analysis, we excluded inbred (F_ROH_ > 0.0625) and hybrid wild boar (F_hybrid_ > 0.0625) from these analyses (see Fig. S5 for the impact on the PCoA). ADMIXTURE 1.3.0 tool assesses the most likely number of genetic clusters and subsequently the individual cluster membership proportions for each individual by means of a maximum likelihood approach. To decrease the bias of uneven sampling on ordination (McVean 2009; DeGiorgio and Rosenberg 2013), we randomly selected a maximum of five individuals per sampling location for the PCoA.

Lastly, to examine spatial patterns, we calculated and mapped multilocus observed heterozygosity (MLH) using R base functions. As a further exploration of patterns of gene flow, we examined allelic clines in European wild boar. We visualized the spatial distribution of the mean frequency of alleles typical for focal regions (selection of SNP loci for which MAF within the focal region is larger than 0.5). We then interpolated the mean allele frequencies over the map of Europe (see Fig. S6 for the spatial distribution of the wild boar samples used for interpolation), using the function *autoKrige* from the R package automap 1.0–14 (Hiemstra et al. 2009).

## Results

### Hybridization and inbreeding

**Fig. 1:**
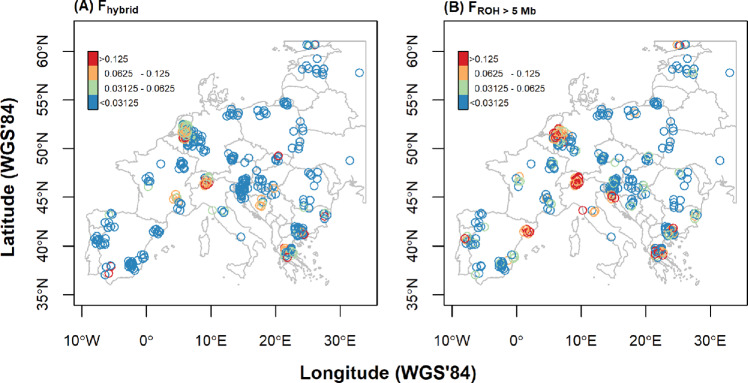
Recent anthropogenic impact on European wild boar. **A** Recent pig hybridization. F_hybrid_ is the fraction of the genome of domestic pig origin, as determined by PCOA projection. F_hybrid_ of 0.250, 0.125, 0.0625 are the expected values for a 2nd, 3rd and 4th generation hybrid. **B** Recent inbreeding. F_ROH > 5 Mb_ is the proportion of the genome that contains Runs of Homozygosity larger than 5 Mb. ROHs longer than 5 Mb stem from common ancestors typically less than 10 generations ago. To reduce the overlap of samples from the same population, the sample locations are jittered. F_ROH_ values of 0.125, 0.0625 and 0.03125 are the expected values for offspring of half siblings, full cousins and half cousins. Please note, however, that these values can also be obtained through accumulation of inbreeding via multiple, distant common ancestors.

**Fig. 2:**
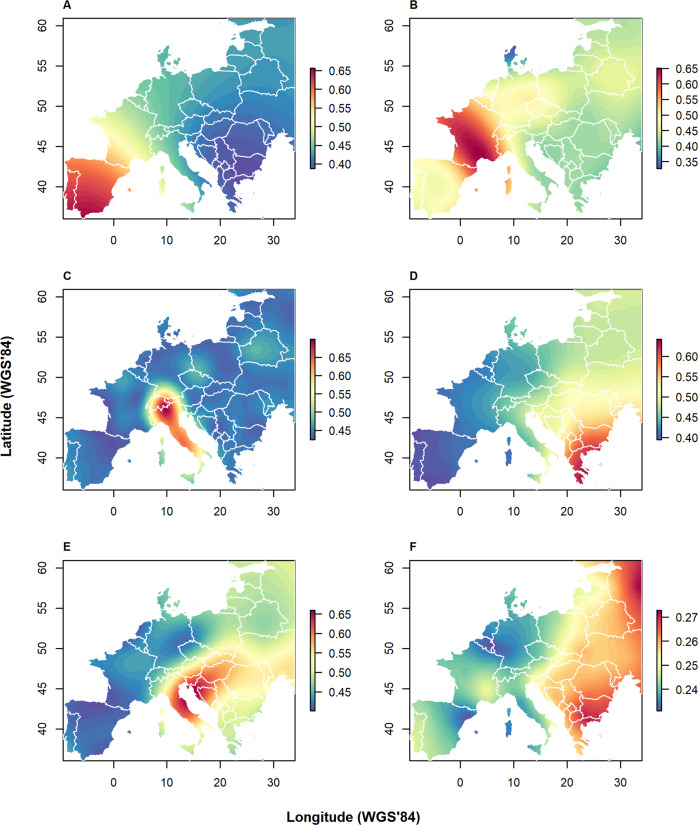
Allelic clines in European wild boar. Spatial distribution of the mean frequency of alleles typical for focal regions (selection of SNP loci for which Minor Allele Frequency (MAF) within the focal region is larger than 0.5): (**A**) the Iberian Peninsula; (**B**) southern France; (**C**) the Italian Peninsula; (**D**) southeastern Europe; (**E**) northwestern Balkans; and (**F**) the Far East. The spatial distribution of the samples underlying the interpolations is shown in Fig. S6. Color scales are calibrated for each region specific selection set of SNP loci.

A spatial map of the projection scores of samples along the axis discriminating between wild boar and pigs in the PCoA showed that wild boar x pig hybrids occur in multiple countries across the continent (Fig. 1A). Using the PCoA projections as a proxy for pig ancestry (F_hybrid_) of the 330 unrelated European wild boar investigated, we estimated that 22 (7%), 20 (3%) and 6 (2%) samples had F_hybrid_ values of 0.063–0.125, 0.125–0.250, and >0.250, respectively. These putative hybrids stemmed mainly from wild boar populations in northwestern Europe, southern Switzerland, Italy, and the Balkans. This result was in line with the observed occurrence of clusters of alleles abundant in pigs, but rare in European wild boar, in the genomes of the putative hybrids (Fig. S3). The median PCoA scores for Iberian, Italian and French wild boar were 0.15, 0.08 and 0.10, respectively (Fig. S5), indicating a high genetic similarity with domestic pigs, whereas in the Balkans and eastern Europe we additionally observed an influence from Asiatic lineages (Fig. 2).

We detected ROHs longer than 5 Mb (inbreeding event < 10 generations ago) in populations all across the continent (Fig. 1B) over a total of 662 Mb scanned regions, equal to 29% of the wild boar autosomal genome (NCBI 2018). In 36 (11%) of the 330 sampled unrelated wild boar, F_ROH>5Mb_ was larger than 0.125. Additionally, 47 wild boar had F_ROH>5Mb_ between 0.063 and 0.125. Most of these inbred individuals were found in just a few sampling locations, namely the populations of northeastern Spain, The Netherlands, western Germany, southern Switzerland and Greece.

### Spatial genetic structure

**Fig. 3:**
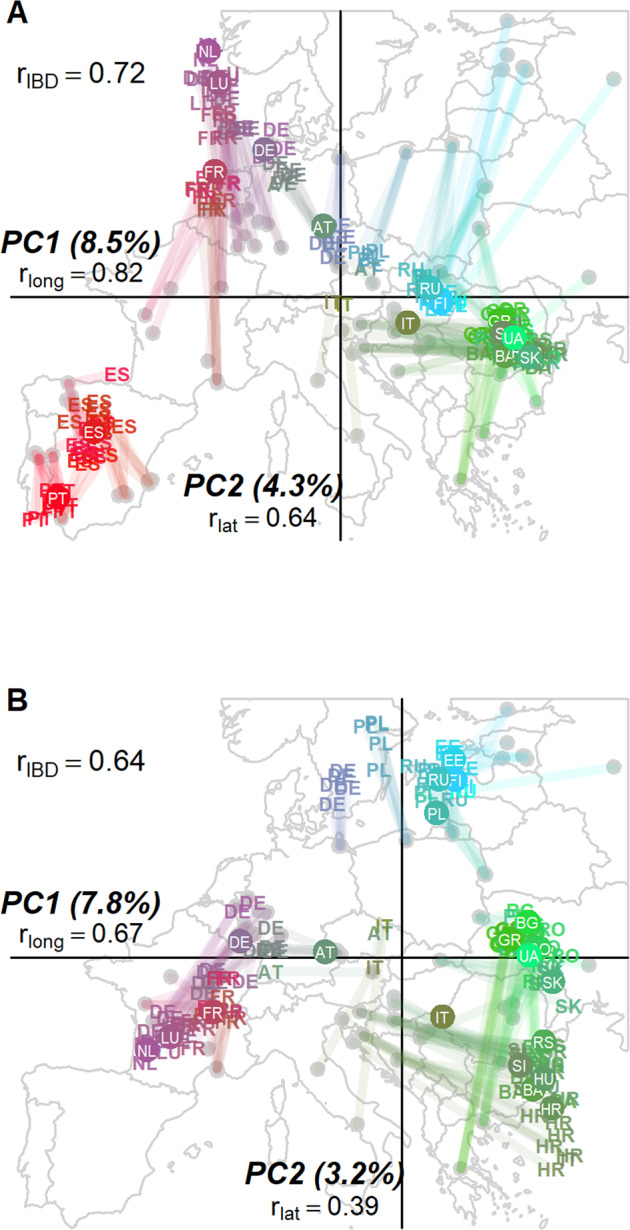
The population genetic structure of European wild boar. PCoA biplots of Hamming genetic distances among wild boar, superimposed on the map of Europe, for wild boar samples of (**A**) the whole European mainland, and (**B**) the European mainland without the Iberian Peninsula. Inbred and hybrid wild boar are excluded. The PCoA scores of the samples are shown as country abbreviations. Colors, lines and dots indicate geographic origin of samples. The shorter the lines, the stronger the match between the PCoA biplot and geography. Filled circles with country codes are the mean PCoA scores per country. rIBD is the Mantel r correlation coefficient of genetic vs. geographic distance. ‘rlong’ and ‘rlat’ are the Spearman rank correlation coefficients of the PCoA first and second axes scores with longitude and latitude, respectively, calculated at population level to reduce autocorrelation.

The first axis of the PCoA of European wild boar distinguished those from western and eastern Europe (Fig. 3A). On the second axis, wild boar from the Iberian peninsula were separated from the rest of western Europe. A subsequent PCoA, without Iberian wild boar, showed, again, separation of western and eastern European wild boar. The second axis distinguished wild boar of northeastern Europe (northeastern Germany, northern Poland, Estonia, Finland and Russia – Kaliningrad region and Central Federal District) from the rest of Europe (Fig. 3B). In both PCoA analyses, wild boar from Italy had a central position, near the origin of the axes, showing that the main axes could not explain the genetic variation of the Italian peninsula. Concordantly, ADMIXTURE analysis could not assign Italian wild boar to any cluster at K = 5, the most likely number of clusters (Fig. S7).

The first and second axes of both PCoAs were correlated with longitude and latitude, respectively (Spearman rank correlation, all *p* < 0.05, whether with or without the Iberian peninsula, see Fig. 3). In addition, there was a significant and strong correlation between geographic and genetic distance of wild boar populations (r_mantel_ = 0.73 and r_mantel_ = 0.63 with and without Iberian peninsula, respectively; both analyses: *p* < 0.001, see Fig. 3). Nevertheless, the clusters detected by PCoA (and supported by ADMIXTURE, Fig. S7) showed deviations from an isolation by distance (IBD) scenario. The first divide detected was the one between Iberian and non-Iberian wild boar (Fig. 3A), but Fig. 3B showed also a major genetic discontinuity in central Europe. Whereas wild boar from Slovenia, Hungary and Slovakia were projected on the right of the PCoA axis 1 together with the eastern group (comprising populations from Poland eastwards), nearby wild boar from southeastern Germany clustered with the western group (namely Germany, France, Belgium and the Netherlands). Samples from Austria appeared to be a contact zone between these two European clusters (PCoA projection at the center of the axis, and mixed ADMIXTURE assignment probabilities, Fig. S7). The second axis of the PCoA showed low genetic dissimilarity among distant wild boar populations, with wild boar from Greece and Bulgaria projecting closer to northeastern European populations than animals from the Carpathians or the Dinaric Alps. Overall, our results show the characteristics of a fragmented cline extending from the Dinaric Alps via southeastern Europe and the Carpathians, to northeastern Europe, and, from there, to western Europe and, ultimately, the Iberian peninsula.

**Fig. 4:**
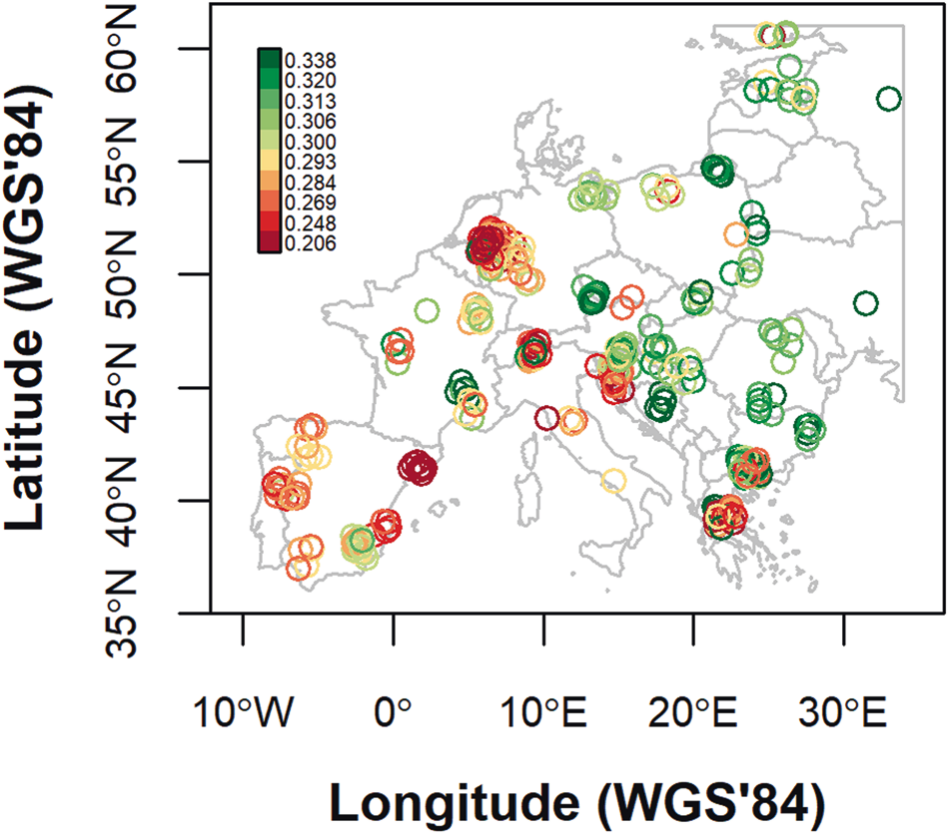
Multilocus Heterozygosity (MLH) of wild boar across Europe. To reduce the overlap of samples from the same population, the sample locations are jittered. MLH values are classified in 10 intervals with an equal number of observations (i.e. deciles). Legend numbers represent the lower boundaries of the intervals.

The spatial distribution of alleles typical for putative refugial areas, or source populations (MAF > 0.5), showed insightful continental-wide patterns (Fig. 2). Alleles typical for the southeastern (Greece and Bulgaria) and the southwestern regions (Iberian peninsula, and southwestern France) gradually decreased in frequency towards northern latitudes (Fig. 2). In contrast, Italian alleles had uniformly low frequency outside the Italian peninsula (Fig. 2). In turn, alleles typical for the Dinaric Alps were relatively abundant in the area south of the Carpathians, present at low frequency in northeastern Europe and absent, or nearly so, in western Europe (Fig. 2E). Lastly, alleles typical for Far East Asia appeared to be relatively abundant in eastern Europe, and in particular in southeastern Europe. There was a clear divide in the allele frequency; to the west of the line extending from the Alps to the Baltic states, including the Italian peninsula, the frequency of alleles typical for wild boar of Far Eastern Asia was markedly lower than in eastern Europe (Fig. 2F).

The spatial map of the Multilocus Heterozygosity (MLH) showed an overall west-east gradient (Fig. 4). We detected a positive correlation between MLH and longitude but no correlation with latitude (general linear model, F = 288.7, r^2^_adj_ = 0.78, d.f. = 324; coefficient estimate longitude = 0.29; t-value estimate longitude = 7.64). Additionally, with the same model, we observed, as expected, a positive correlation between MLH and F_hybrid_ (coefficient estimate = 0.22, t-value estimate = 12.1) and a strongly negative correlation with F_ROH_ (coefficient estimate = −0.56, t-value estimate = −31.67). All general linear models had *p* values < 0.001.

## Discussion

Using genome-wide data and a continent-wide coverage of populations across Europe, we examined recent anthropogenic impact on, and revealed the spatial structure of, the genetic variation of European wild boar. We found considerable and widespread anthropogenic impact on wild boar in the form of inbreeding, fragmentation and pig introgression, though not translocation. Nevertheless, continent-wide PCoA analysis, ADMIXTURE analysis and spatial maps of allele frequencies and heterozygosity showed that the genetic composition of wild boar in Europe takes the form of an arch-shaped cline that extends from southeastern Europe, via the Baltic region, to western Europe and, finally, the Iberian peninsula – the latter being much differentiated from the rest of the continent. Lastly, we found an increasing gradient of heterozygosity from west to east, with the highest levels of heterozygosity found in a line extending from the Alps to the Baltic region.

These observations stimulate the ongoing discussion on what shapes the genetic structure of European wild boar, and northern hemisphere wildlife in general. They are partially concordant with previous studies based on mtDNA (Scandura et al. 2011a; Alexandri et al. 2012; Kusza et al. 2014; Vilaça et al. 2014; Veličković et al. 2015; Maselli et al. 2016) and microsatellites (Vernesi et al. 2003; Scandura et al. 2008; Veličković et al. 2016), that reported a gradient in genetic variability and different contributions from glacial refugia. However, our findings additionally show unreported patterns highlighting the relative importance of (i) anthropogenic influences versus natural processes, and (ii) the LGM versus other natural processes. We therefore argue that the genetic structure of wild boar can only be understood from a complex, multi-faceted perspective, that integrates many different and sometimes opposing demographic processes. To aid understanding, our inferences—summarized in Table S1 and introduced and explained below—are spatially conceptualized in Fig. S8.

### Anthropogenic influences

Frequent and strong signatures of inbreeding and pig hybridization indicate a recent anthropogenic impact on the genetic variation of European wild boar. Earlier, *inter alia* Goedbloed et al. (2013a) provided evidence for the occurrence of pig hybridization at regional scale, which leads to a genetic signature of aberrant genomic segments with unusual alleles that are indicative of introgressed haplotypes (Goedbloed et al. 2013a). Our findings identified that recent hybridization, although geographically confined, occurred in several countries. However, introgressed pig haplotypes can only be detected if the wild and domestic populations are two separate gene pools. The high observed genetic similarity of Iberian and Italian wild boar with domestic pigs (see Fig. S2) was difficult to ascertain through detection of introgressed haplotypes. Either the signal is a type-I error and pig hybridization is infrequent (in line with findings for Italian wild boar by Scandura et al. 2022) or pig hybridization has been so frequent and long-lasting that introgressed segments are too short to detect with medium density SNP data. Both Iberia and Italy share a long tradition of extensive pig herding for regional ham production, up to the present, that possibly has led to a prolonged gene-flow between the wild boar and domestic pigs (Herrero-Medrano et al. 2013; Canu et al. 2014; Iacolina et al. 2016; Maselli et al. 2016). Pig hybridization may perhaps even partially explain the observed genetic differentiation of the southern peninsulas, in particular Iberia, from the rest of the European continent (next to natural barriers effect, such of the Pyrenees mountains, to be discussed below).

The presence of local genetic discontinuities in combination with many, long ROH segments is likely the genetic legacy of size reductions and fragmentation of wild boar populations. Earlier work showed genetic fragmentation in specific regions (e.g., Scandura et al. 2008; Ferreira et al. 2009; Goedbloed et al. 2013b; Kusza et al. 2014); the present study indicates that fragmentation is a continent-wide phenomenon that appears to be most eminent in areas of high human population density. We observed genetic discontinuities in western and central Europe and among the western, northeastern, Carpathian, Dinaric and southeastern clusters. The separation of the northeastern cluster coincides with the near absence of wild boar in northwestern Europe (northern Netherlands and northwestern Germany; Goedbloed et al. 2013b) and southeastern Poland at the start of the 20^th^ century (Apollonio et al. 2010). In eastern and southeastern Europe, genetic discontinuities along the Galicia, Pannonian and Dacian basins are associated with human presence. These fertile river floodplains are, and have been, densely inhabited and the landscape has been altered into agricultural land, with only small patches of forests. This change in land use, combined with overhunting, led to low wild boar densities during the 19^th^ and (first half of the) 20^th^ centuries. Our results appear to still reflect this situation, despite a recent comeback of the species in those areas (Apollonio et al. 2010; Deinet et al. 2013). The erratic spatial pattern in inbreeding (as determined through F_ROH_) indicates that the severity of fragmentation and associated population size reductions vary from region to region. The populations with the highest levels of inbreeding (e.g., Collserola, Northeast Spain; Meinweg, Southeast Netherlands) share a history of strong isolation and severe bottlenecks. For all these populations, the bottlenecks mostly occurred approximately one or half a century ago. Although these populations are currently increasing and might keep doing so in the future, coancestry and hence inbreeding may continue to accumulate (see e.g., Kardos et al. 2018).

What are the potential phenotypic and ecological consequences of these human-caused alterations of genetic variation? Apart from potentially lowering adaptive potential, the observed levels of inbreeding have in other wildlife species been shown to be associated with body weight and juvenile survival (Bérénos et al. 2016; Huisman et al. 2016). For wild boar specifically, there are however, to our best knowledge, no reported cases of inbreeding depression. In contrast, pig hybridization is in the field often inferred by aberrant phenotypic variation, in particular multicolored coats. Genetically, pig hybridization might cause introgression of selected haplotypes in small regions of the genome (Ai et al. 2015) over time. Although limited to few and small regions of the genome, these haplotypes might cause long-term phenotypic consequences (Canu et al. 2016; Fulgione et al. 2016; Iacolina et al. 2019), including vital phenotypic traits such as litter size (Fulgione et al. 2016).

### Spatial genetic structure: Anthropogenic influences *vs.* natural processes

Whilst frequent and strong, the anthropogenic-caused alterations of genetic variation do not appear to have erased the continent-wide spatial genetic patterns of natural origin. The two main continent-wide genetic patterns observed were an arch shaped genetic cline (extending from western to northeastern and southeastern Europe), and a complex heterozygosity gradient (low in the west, high in the east, the highest in the line from the Alps to the Baltic region). Assuming that anthropogenic influences typically produce erratic patterns (see e.g., De Jong et al. 2020), we suspect that the regularity of these patterns imply underlying forces of natural origin. This regularity is highlighted by the fact that IBD proved to be a strong predictor in our results.

Several apparently contradictory patterns emerge from our results: (i) multiple local genetic discontinuities despite the existence of a strong pattern of isolation by distance; (ii) severe inbreeding occurring locally, despite a continental-wide heterozygosity gradient; (iii) differentiation patterns correlated with longitude and latitude, despite substantial pig hybridization. These seemingly contradictory results are likely explained by scale and resolution as large-scale spatial genetic structure may be insensitive to local alterations of genetic variation. Hence, the question on to what extent human activities alter genetic variation of wildlife, is much dependent on spatial scale (see, for example, the continental-wide study of Scandura et al. (2008) and the regional study of Ferreira et al. (2009)). Furthermore, even when genetic alteration may be negligible in a whole genome perspective, the phenotypic and ecological impact may be considerable (such as e.g., an increase in litter size; Fulgione et al. (2016)). Human alteration of genetic variation of wildlife should thus perhaps be evaluated at the phenotypic scale.

### Spatial genetic structure: LGM *vs*. other natural processes

The existence of refugia during the LGM, and the southwards retraction and northwards recolonization from these refugia, is generally considered to be the most dominant natural force shaping genetic variation of European wildlife (Hewitt 1999). In two ways, our findings may give understanding of the genetic legacy of the LGM: (i) the location of a suture zone (i.e., area of secondary contact), and (ii) the source populations for the post-LGM northward recolonization. High levels of heterozygosity along the line Alps-Baltics (Fig. 4), and in addition a sharp transition of allele frequencies over this line (Fig. 2) may indicate that this is a suture zone, i.e., an area of secondary contact between two divergent source populations. This observation is further supported by the correspondence with the known transition of the western karyotype of 2n = 36 to the ancestral eastern karyotype of 2n = 38 (Rejduch et al. 2003; Fang et al. 2006). However, we could not exclude that the genetic discontinuity in central Europe is a consequence of local eradications and subsequent recovery (or reintroductions) of wild boar since the second world war, as discussed above (Krže 1982; Deinet et al. 2013; Bragina et al. 2015).

Accepting the idea of a western and an eastern clade, based on our results we infer a pivotal role of southern France as a western post-glacial expansion source, concordantly with previous genetic and archeological studies (Sommer and Nadachowski 2006; Sommer and Zachos 2009; Vilaça et al. 2014), whereas contributions from the Iberian and Italian peninsulas are unlikely. Although, in agreement with previous studies (Scandura et al. 2011a; Alexandri et al. 2012; Kusza et al. 2014; Vilaça et al. 2014; Veličković et al. 2015; Maselli et al. 2016), the Iberian and Italian peninsulas showed signs of differentiation compatible with glacial refugia, our results differ in terms of their contribution to post-glacial recolonization patterns. The heterogeneous but still distinct genetic characteristics of the Italian population suggests a minor role in post-glacial colonization patterns, which is in contrast with previous studies (Alexandri et al. 2012; Vilaça et al. 2014; Veličković et al. 2015, 2016, but see Hewitt 1999). While we may possibly have failed to detect a contribution of Italian wild boar in post-glacial recolonization of Europe, due to the relatively small sample size, a negligible contribution of the Italian peninsula matches patterns inferred in other European mammals (Taberlet et al. 1998; Hewitt 1999), including roe (*Capreolus capreolus*; Lorenzini et al. 2002) and red deer (Sommer et al. 2009). Additionally, the Iberian peninsula - with its marked genetic differentiation from the rest of the continent – also appears to have played a negligible role during the recolonization process, which is concordant with results from Vilaça et al. (2014) and observed in other species as well (roe deer, Lorenzini et al. 2003; red deer, Carranza et al. 2016).

In the east, northward colonization likely took place from the Balkans with multiple refugia (i.e., Dinaric Alps, the Carpathians and Slavyanka mountains), as previously suggested (Alexandri et al. 2012, 2017; Veličković et al. 2015, 2016) and in agreement with environmental simulations (Vilaça et al. 2014). However, the spatial distribution of allelic frequencies showed a non-neglectable contribution of eastern lineages to eastern European populations, contrary to what has previously been reported (Manunza et al. 2013), suggesting a possible colonization route from the Caucasus (Schmitt and Varga 2012). This eastern component might contribute to explaining why northeastern Europe is genetically more similar to southeastern Europe than to the nearby Carpathians and the higher variability in eastern Europe compared to the southern populations.

The formation of the suture zone on the northeastward line Alps-Baltics rather than a northward line, might have been caused by an earlier, or faster, expansion by wild boar from southern France, that consequently recolonized most of northern Europe. A similar pattern is suggested for red deer by fossil records and microsatellite variation (Sommer et al. 2008; Zachos et al. 2016). An explanation may be that at the start of the Holocene (in particular, around 9000 years ago) the region south of the Tatras Mountains was still relatively cold and dominated by coniferous trees, while in western Europe temperatures were milder and broadleaved forests dominated the vegetation (Brewer et al. 2002; Cheddadi and Bar-Hen 2009). The absence of autumn mast and occurrence of severe winters likely limited the survival of wild boar in southeastern Europe during the early Holocene, similar to boreal forests of northern Europe today (Melis et al. 2006; Apollonio et al. 2010).

Next to assumed LGM signatures, we also observed spatial genetic patterns not easily reconcilable with the LGM impact. First and foremost, the PCoA (Fig. 3) showed an ordination that is atypical for a postglacial expansion. Due to allele surfing (Braga et al. 2019) and the consequential genetic drift, a PCoA on expanding populations typically opposes individuals of recolonized areas, not refugial areas (Franois et al. 2010). The fact that our PCoA found the largest genetic differences among southern populations, rather than northern ones, shows that the signature of the LGM has become relatively weak in the autosomal genome. Second, except for a circumstantial bottleneck in the western refugium, there is no known LGM mechanism that could have caused low heterozygosity in western Europe. Instead, the central–marginal hypothesis may apply, which holds that in the core of the distribution, which for wild boar is Asia, there is a higher genetic diversity than in the periphery (Eckert et al. 2008). Arguably, the immigration history from Asia towards Europe (Azzaroli et al. 1988) and particularly western Europe, the periphery, is a more likely explanation of the observed heterozygosity cline. Such immigration has commenced in the Middle Pleistocene and might have occurred repeatedly throughout the Lower Pleistocene and Holocene (Palombo and Romana 2003; Magri 2013). Ultimately, this implies that the LGM as dominant, leave alone, sole natural force shaping the genetic structure of wild boar–and other northern hemisphere wildlife – may be too simple a picture.

## Conclusions

We provided evidence that the spatial genetic variation of European wild boar is the outcome of a complex interplay of multiple processes of both anthropogenic and natural origin. We observed many and strong signatures that we attribute to human impacts, including loss of genetic variation due to inbreeding, increased genetic variation due to pig introgression, and the existence of genetic discontinuities in areas without natural barriers on the background of a continent-wide pattern dating back to the LGM, or possibly even earlier. Future research, involving historical samples and sequence data should study the origin of haplotypes to shed light on the diverse ancestry of wild boar, and other species, of the European continent, while modern samples should be investigated to identify how anthropogenic influences are affecting the survival and adaptability of the species, also considering the need of science-based management practices for a species that is increasingly considered a source of human-wildlife conflict.

## Supplementary information

Supplementary tables

Supplementary figures

## Data Availability

Genotypes analysed in this article are available on Dryad 10.5061/dryad.1rn8pk0z6.

## References

[CR1] Ai H, Fang X, Yang B, Huang Z, Chen H, Mao L (2015). Adaptation and possible ancient interspecies introgression in pigs identified by whole-genome sequencing. Nat Genet.

[CR2] Alexander DH, Novembre J, Lange K (2009). Fast model-based estimation of ancestry in unrelated individuals. Genome Res.

[CR3] Alexandri P, Megens HJ, Crooijmans RPMA, Groenen MAM, Goedbloed DJ, Herrero-Medrano JM (2017). Distinguishing migration events of different timing for wild boar in the Balkans. J Biogeogr.

[CR4] Alexandri P, Triantafyllidis A, Papakostas S, Chatzinikos E, Platis P, Papageorgiou N (2012). The Balkans and the colonization of Europe: the post-glacial range expansion of the wild boar, *Sus scrofa*. J Biogeogr.

[CR5] Alves PC, Pinheiro I, Godinho R, Vicente JJ, Gortázar C, Scandura M (2010). Genetic diversity of wild boar populations and domestic pig breeds (*Sus scrofa*) in South-western Europe. Biol J Linn Soc.

[CR6] Apollonio M, Andersen R, Putman R (2010) *European ungulates and their management in the 21st century* (M Apollonio, R Andersen, and R Putman, Eds.) Cambridge University Press: Cambridge, UK

[CR7] Azzaroli A, De Giuli C, Ficcarelli G, Torre D (1988). Late pliocene to early mid-pleistocene mammals in Eurasia: Faunal succession and dispersal events. Palaeogeogr Palaeoclimatol Palaeoecol.

[CR8] Bérénos C, Ellis PA, Pilkington JG, Pemberton JM (2016). Genomic analysis reveals depression due to both individual and maternal inbreeding in a free‐living mammal population. Mol Ecol.

[CR9] Braga RT, Rodrigues JFM, Diniz-Filho JAF, Rangel TF (2019). Genetic population structure and allele surfing during range expansion in dynamic habitats. An da Academia Brasileira de Ciências.

[CR10] Bragina EV, Ives AR, Pidgeon AM, Kuemmerle T, Baskin LM, Gubar YP, Piquer-Rodríguez M, Keuler NS, Petrosyan VG, Radeloff VC (2015). Rapid Declines of Large Mammal Populations after the Collapse of the Soviet Union. Cons Biol.

[CR11] Brewer S, Cheddadi R, de Beaulieu JL, Reille M, Allen J, Almqvist-Jacobson H (2002). The spread of deciduous *Quercus* throughout Europe since the last glacial period. For Ecol Manag.

[CR12] Cahill S, Llimona F, Cabañeros L, Calomardo F (2012). Characteristics of wild boar (*Sus scrofa*) habituation to urban areas in the Collserola Natural Park (Barcelona) and comparison with other locations. Anim Biodivers Conserv.

[CR13] Canu A, Costa S, Iacolina L, Piatti P, Apollonio M, Scandura M (2014). Are captive wild boar more introgressed than free-ranging wild boar? Two case studies in Italy. Eur J Wildl Res.

[CR14] Canu A, Vilaça STT, Iacolina L, Apollonio M, Bertorelle G, Scandura M (2016). Lack of polymorphism at the MC1R wild-type allele and evidence of domestic allele introgression across European wild boar populations. Mamm Biol.

[CR15] Carranza J, Salinas M, de Andrés D, Pérez-González J (2016). Iberian red deer: paraphyletic nature at mtDNA but nuclear markers support its genetic identity. Ecol Evol.

[CR16] Chang CC, Chow CC, Tellier LCAM, Vattikuti S, Purcell SM, Lee JJ (2015). Second-generation PLINK: Rising to the challenge of larger and richer datasets. Gigascience.

[CR17] Cheddadi R, Bar-Hen A (2009). Spatial gradient of temperature and potential vegetation feedback across Europe during the late Quaternary. Clim Dyn.

[CR18] Clark PU, Dyke AS, Shakun JD, Carlson AE, Clark J, Wohlfarth B (2009). The Last Glacial Maximum. Science.

[CR19] DeGiorgio M, Rosenberg NA (2013). Geographic sampling scheme as a determinant of the major axis of genetic variation in principal components analysis. Mol Biol Evol.

[CR20] Deinet S, Ieronymidou C, McRae L, Burfield IJ, Foppen RP, Collen B, et al. (2013) *Wildlife comeback in Europe. The recovery of selected mammal and bird species*. London, UK

[CR21] Eckert CG, Samis KE, Lougheed SC (2008). Genetic variation across species’ geographical ranges: the central–marginal hypothesis and beyond. Mol Ecol.

[CR22] Fang M, Berg F, Ducos A, Andersson L (2006). Mitochondrial haplotypes of European wild boars with 2n = 36 are closely related to those of European domestic pigs with 2n = 38. Anim Genet.

[CR23] Ferenčaković M, Sölkner J, Curik I (2013). Estimating autozygosity from high-throughput information: Effects of SNP density and genotyping errors. Genet Sel Evol.

[CR24] Ferreira E, Souto L, Soares AMVM, Fonseca C (2009). Genetic structure of the wild boar population in Portugal: Evidence of a recent bottleneck. Mamm Biol.

[CR25] Franois O, Currat M, Ray N, Han E, Excoffier L, Novembre J (2010). Principal component analysis under population genetic models of range expansion and admixture. Mol Biol Evol.

[CR26] Frantz AC, Bertouille S, Eloy MC, Licoppe A, Chaumont F, Flamand MC (2012). Comparative landscape genetic analyses show a Belgian motorway to be a gene flow barrier for red deer (*Cervus elaphus*), but not wild boars (*Sus scrofa*). Mol Ecol.

[CR27] Fulgione D, Rippa D, Buglione M, Trapanese M, Petrelli S, Maselli V (2016). Unexpected but welcome. Artificially selected traits may increase fitness in wild boar. Evol Appl.

[CR28] Goedbloed DJ, Megens HJ, van Hooft P, Herrero-Medrano JM, Lutz W, Alexandri P (2013). Genome-wide single nucleotide polymorphism analysis reveals recent genetic introgression from domestic pigs into Northwest European wild boar populations. Mol Ecol.

[CR29] Goedbloed DJ, van Hooft P, Megens HJ, Langenbeck K, Lutz W, Crooijmans RPMA (2013). Reintroductions and genetic introgression from domestic pigs have shaped the genetic population structure of Northwest European wild boar. BMC Genet.

[CR30] Groenen MAM, Archibald AL, Uenishi H, Tuggle CK, Takeuchi Y, Rothschild MF (2012). Analyses of pig genomes provide insight into porcine demography and evolution. Nature.

[CR31] Herrero-Medrano JM, Megens H-J, Groenen MAM, Ramis G, Bosse M, Pérez-Enciso M (2013). Conservation genomic analysis of domestic and wild pig populations from the Iberian Peninsula. BMC Genet.

[CR32] Hewitt GM (1999). Post-glacial re-colonization of European biota. Biol J Linn Soc.

[CR33] Hewitt GM (2004). Genetic consequences of climatic oscillations in the Quaternary. Philos Trans R Soc Lond Ser B Biol Sci.

[CR34] Hiemstra PH, Pebesma EJ, Twenhöfel CJW, Heuvelink GBM (2009). Real-time automatic interpolation of ambient gamma dose rates from the Dutch radioactivity monitoring network. Comput Geosci.

[CR35] Howrigan DP, Simonson MA, Keller MC (2011). Detecting autozygosity through runs of homozygosity: a comparison of three autozygosity detection algorithms. BMC Genomics.

[CR36] Huisman J, Kruuk LEB, Ellis PA, Clutton-Brock T, Pemberton JM (2016). Inbreeding depression across the lifespan in a wild mammal population. Proc Natl Acad Sci.

[CR37] Iacolina L, Corlatti L, Buzan E, Safner T, Šprem N (2019). Hybridisation in European ungulates: an overview of the current status, causes, and consequences. Mamm Rev.

[CR38] Iacolina L, Pertoldi C, Amills M, Kusza S, Megens H-J, Bâlteanu VA (2018). Hotspots of recent hybridization between pigs and wild boars in Europe. Sci Rep..

[CR39] Iacolina L, Scandura M, Goedbloed DJ, Alexandri P, Crooijmans RPMA, Larson G (2016). Genomic diversity and differentiation of a managed island wild boar population. Heredity.

[CR40] Jombart T, Ahmed I (2011). adegenet 1.3-1: new tools for the analysis of genome-wide SNP data. Bioinformatics.

[CR41] de Jong JF, Hooft van P, Megens HJ, Crooijmans RPMA, Groot de GA, Pemberton JM, Huisman J (2020). Fragmentation and translocation distort the genetic landscape of ungulates: red deer in the Netherlands. Front Ecol Evol.

[CR42] Kamvar ZN, Tabima JF, Grünwald NJ (2014). *Poppr*: an R package for genetic analysis of populations with clonal, partially clonal, and/or sexual reproduction. PeerJ.

[CR43] Kardos M, Åkesson M, Fountain T, Flagstad Ø, Liberg O, Olason P (2018). Genomic consequences of intensive inbreeding in an isolated wolf population. Nat Ecol Evol.

[CR44] Kaplan JO, Krumhardt KM, Zimmermann N (2009) The prehistoric and preindustrial deforestation of Europe. Quat Sci Rev 28:3016–3034. 10.1016/j.quascirev.2009.09.028

[CR45] Koemle D, Zinngrebe Y, Yu X (2018). Highway construction and wildlife populations: Evidence from Austria. Land use policy.

[CR46] Krže B (1982). Divji prašič: biologija, gojitev, ekologija.

[CR47] Kusza S, Podgórski T, Scandura M, Borowik T, Jávor A, Sidorovich VE (2014). Contemporary genetic structure, phylogeography and past demographic processes of wild boar *Sus scrofa* population in central and eastern Europe. PLoS One.

[CR48] Lorenzini R, Lovari S, Masseti M (2002). The rediscovery of the Italian roe deer: Genetic differentiation and management implications. Ital J Zool.

[CR49] Lorenzini R, San José C, Braza F, Aragón S (2003). Genetic differentiation and phylogeography of roe deer in Spain, as suggested by mitochondrial DNA and microsatellite analysis. Ital J Zool.

[CR50] Magri D (2013). Early to Middle Pleistocene dynamics of plant and mammal communities in South West Europe. Quat Int.

[CR51] Manunza A, Zidi A, Yeghoyan S, Balteanu VA, Carsai TC, Scherbakov O (2013). A high throughput genotyping approach reveals distinctive autosomal genetic signatures for European and Near Eastern wild boar. PLoS One.

[CR52] Maselli V, Rippa D, De Luca A, Larson G, Wilkens B, Linderholm A (2016). Southern Italian wild boar population, hotspot of genetic diversity. Hystrix.

[CR53] McVean G (2009). A genealogical interpretation of principal components analysis. PLoS Genet.

[CR54] Megens H-J, Crooijmans RP, Cristobal M, Hui X, Li N, Groenen MA (2008). Biodiversity of pig breeds from China and Europe estimated from pooled DNA samples: differences in microsatellite variation between two areas of domestication. Genet Sel Evol.

[CR55] Melis C, Szafrańska PA, Jȩdrzejewska B, Bartoń K (2006). Biogeographical variation in the population density of wild boar (*Sus scrofa*) in western Eurasia. J Biogeogr.

[CR56] Mihalik B, Stéger V, Frank K, Szendrei L, Kusza S (2018). Barrier effect of the M3 highway in Hungary on the genetic diversity of wild boar (*Sus scrofa*) population. Res J Biotechnol.

[CR57] NCBI (2018) Genome Organism Overview: *Sus scrofa* (pig). https://www.ncbi.nlm.nih.gov/genome?term=sus%20scrofa%20%5BOrganism%5D&cmd=DetailsSearch&report=Overview

[CR58] Nikolov IS, Gum B, Markov G, Kuehn R (2009). Population genetic structure of wild boar *Sus scrofa* in Bulgaria as revealed by microsatellite analysis. Acta Theriol (Warsz).

[CR59] Nykänen M, Rogan E, Foote AD, Kaschner K, Dabin W, Louis M (2019). Postglacial colonization of northern coastal habitat by bottlenose dolphins: a marine leading-edge expansion?. J Hered.

[CR60] Palombo M, Romana AV-G (2003) Remarks on the biochronology of mammalian faunal complexes from the Pliocene to the Middle Pleistocene in France. Geol Rom: 145–163

[CR61] Paradis E, Claude J, Strimmer K (2004). APE: analysis of phylogenetics and evolution in R language. Bioinformatics.

[CR62] Purcell S, Neale B, Todd-Brown K, Thomas L, Ferreira MAR, Bender D et al (2007) PLINK: A tool Set for whole-genome association and population-based linkage analyses. Am J Hum Genet 81:559–575. www.cog-genomics.org/plink/1.9/10.1086/519795 PMC1950838 17701901

[CR63] Putman R, Apollonio M, Andersen R (2011). Ungulate management in Europe: problems and practices.

[CR64] R Core Team (2018) *R: A language and environment for statistical computing*. Vienna, Austria

[CR65] Rejduch B, Sota E, Ró M, Ko M (2003). Chromosome number polymorphism in a litter of European wild boar (*Sus scrofa scrofa* L.). Anim Sci Pap Rep..

[CR66] Scandura M, Iacolina L, Apollonio M (2011). Genetic diversity in the European wild boar *Sus scrofa*: phylogeography, population structure and wild x domestic hybridization: Genetic variation in European wild boar. Mamm Rev.

[CR67] Scandura M, Iacolina L, Cossu A, Apollonio M (2011). Effects of human perturbation on the genetic make-up of an island population: The case of the Sardinian wild boar. Heredity.

[CR68] Scandura M, Iacolina L, Crestanello B, Pecchioli E, Di Benedetto MF, Russo V (2008). Ancient vs. recent processes as factors shaping the genetic variation of the European wild boar: Are the effects of the last glaciation still detectable?. Mol Ecol.

[CR69] Scandura M, Fabbri G, Caniglia R, Iacolina L, Mattucci F, Mengoni C, Pante G, Apollonio M, Mucci N (2022). Resilience to Historical Human Manipulations in the Genomic Variation of Italian Wild Boar Populations. Front Ecol Evol.

[CR70] Schmitt T, Varga Z (2012). Extra-Mediterranean refugia: the rule and not the exception. Front Zool.

[CR71] Sommer RS, Fahlke JM, Schmölcke U, Benecke N, Zachos FE (2009). Quaternary history of the European roe deer Capreolus capreolus. Mamm Rev.

[CR72] Sommer RS, Nadachowski A (2006). Glacial refugia of mammals in Europe: evidence from fossil records. Mamm Rev.

[CR73] Sommer RS, Zachos FE (2009). Fossil evidence and phylogeography of temperate species: ‘glacial refugia’ and post-glacial recolonization. J Biogeogr.

[CR74] Sommer RS, Zachos FE, Street M, Jöris O, Skog A, Benecke N (2008). Late Quaternary distribution dynamics and phylogeography of the red deer (*Cervus elaphus*) in Europe. Quat Sci Rev.

[CR75] Stillfried M, Fickel J, Börner K, Wittstatt U, Heddergott M, Ortmann S (2017). Do cities represent sources, sinks or isolated islands for urban wild boar population structure?. J Appl Ecol.

[CR76] Taberlet P, Fumagalli L, Wust-Saucy AG, Cossons JF (1998). Comparative phylogeography and post-glacial colonization routes in Europe. Mol Ecol.

[CR77] Veličković N, Djan M, Ferreira E, Stergar M, Obreht D, Maletić V (2015). From north to south and back: the role of the Balkans and other southern peninsulas in the recolonization of Europe by wild boar. J Biogeogr.

[CR78] Veličković N, Ferreira E, Djan M, Ernst M, Obreht Vidaković D, Monaco A (2016). Demographic history, current expansion and future management challenges of wild boar populations in the Balkans and Europe. Heredity.

[CR79] Vernesi C, Crestanello B, Pecchioli E, Tartari D, Caramelli D, Hauffe H (2003). The genetic impact of demographic decline and reintroduction in the wild boar (*Sus scrofa*): A microsatellite analysis. Mol Ecol.

[CR80] Vilaça ST, Biosa D, Zachos F, Iacolina L, Kirschning J, Alves PC (2014). Mitochondrial phylogeography of the European wild boar: The effect of climate on genetic diversity and spatial lineage sorting across Europe. J Biogeogr.

[CR81] Zachos FE, Frantz AC, Kuehn R, Bertouille S, Colyn M, Niedziałkowska M et al. (2016) Genetic structure and effective population sizes in European red deer (*Cervus elaphus*) at a continental scale: insights from microsatellite DNA. J Hered 107:318–326 10.1093/jhered/esw011 PMC4888435 26912909

