# Supplementary material for: Spatial genetic structure of European wild boar, with inferences on late-Pleistocene and Holocene demographic history
Source: Heredity (Edinb). 2023 Jan 13;130(3):135–44. doi: 10.1038/s41437-022-00587-1 (PMC9981775; doi:10.1038/s41437-022-00587-1)
Supplement: Supplementary file 1 — Supplementary tables [file 41437_2022_587_MOESM1_ESM.docx]

**Spatial genetic structure of European wild boar, with inferences on late-Pleistocene and Holocene demographic history**

Joost F. de Jong^1^, Laura Iacolina^2,3*^, Herbert H.T. Prins^4^, Pim van Hooft^1^, Richard P.M.A. Crooijmans^5^, Sip E. van Wieren^1^, Joaquin Vicente Baños^6^, Eric Baubet^7^, Seán Cahill^8^, Eduardo Ferreira^9^, Carlos Fonseca^9^, Peter M. Glazov^10^, Ida Jelenko Turinek^11^, András Náhlik^12,13^, Victor M. Lizana Martín^14,15^, Boštjan Pokorny^16,17^, Tomasz Podgórski^18,19^, Nikica Šprem^20^, Rauno Veeroja^21^, Ronald C. Ydenberg^22^ and Hendrik-Jan Megens^5^

**Table S1 Overview of hypothesized and observed (based on SNP analysis) patterns in the genetic structure of European wild boar.** The blue and red colors indicate concordance and discordance, respectively, with hypotheses.

|  |  | **Process** | **Time period** | **Postulated signature in SNP data** | **Observed signature in SNP data** |
| --- | --- | --- | --- | --- | --- |
| 1 |  | **Anthropogenic gene flow processes** |  |  |  |
|  | a | Pig hybridization | Anthropocene | Genetic similarity with pigs, and therewith disruption of natural landscape spatial genetic patterns (Isolation by Distance, and Isolation by Resistance) | - High genetic similarity of wild boars from the Iberian and Italian peninsula to pigs - In addition, in specific populations across Europe: Signatures of recent introgression, including long pig haplotypes |
|  | b | Translocation | Anthropocene | Genetic similarity with distant rather than nearby wild boar | - Absence of wild boar that are genetically more similar to distant rather than nearby populations |
|  | c | Overexploitation and associated fragmentation | Anthropocene | i. Genetic discontinuities in the absence of, or even in disagreement with, natural physical barriers   ii. Signatures of inbreeding in isolated wild population | i. Genetic discontinuities in, amongst others, the Galician, Pannonian and Dacian basins (with mountains seemingly acting as refugia rather than barrier)  ii. Across Europe, wild boar populations are found that have heterozygosity lower than the regionwide baseline value, which is typically associated with frequent Runs of Homozygosity |
| 2 |  | **Interglacial gene flow processes** Effect of the geography of the European continent | Holocene | i. Isolation by Distance  ii. Isolation by Resistance: Due to natural barriers (e.g. water bodies, mountains), genetic isolation of geographically isolated areas, such as the Iberian and Apennine peninsula | i. Isolation by Distance signal: Correlation between first two PCA axes and longitude and latitude, respectively   ii. Isolation by Resistance signal: - Genetic isolation of wild boar of the Iberian peninsula  - Ambiguous signature of Italian wild boar |
| 3 |  | **Glacial gene flow processes** Range retraction towards and expansion from glacial refugia (in particular: Last Glacial Maximum) | Late Pleistocene | i. Gradient in genetic diversity from refugia (southern areas) to recolonized (northern) areas  ii. Latitudinal oriented genetic clusters, such that recolonized areas are genetically similar to their refugial sources  i & ii. PCA axis opposes recolonized areas (Franois et al. 2010)  iii. Secondary contact suture zone somewhere in Northern Europe, causing a region of high heterozygosity | i. Longitudinal, rather than latitudinal, gradient of heterozygosity    ii. Differentiation of West and Southeast Europe (via Northwest Europe), but within Western-Europe strong separation of Iberia  i & ii. PCA axis opposes southern areas (Iberia vs. Balkan; i.e. the former refugial areas), rather than the recolonized areas  iii. Wild boar populations located on the line Alps to Baltics have a genetic signature in between western and eastern Europe, and also have high heterozygosity. This is, possibly, indicative for a suture zone |
| 4 |  | **Species range gene flow processes** Central-marginal hypothesis | Pleistocene till present | i. Allelic gradients from Asia to West-Europe   ii. Genetic diversity is lower in the periphery (e.g., Western-Europe) than the core of the species range (i.e., Asia) | i. High frequency of typical Asian alleles in eastern, and in particular, south-eastern Europe  ii. Low heterozygosity in western Europe, but high heterozygosity in the line Alps-Baltics, rather than Eastern Europe |

**Table S2** Number of samples per country. A) European wild boar; B) Asian wild boar; C) domestic pig breeds.

A)

| **Country** | **Country (abbreviation)** | **Source** | **N** | **N filter missing-ness** | **N filter relatives** | **N filter hybrids** | **N filter F ROH** |
| --- | --- | --- | --- | --- | --- | --- | --- |
| Austria | Aut | Iacolina et al. 2016 | 2 | 2 | 2 | 2 | 1 |
| Bulgaria | Bgr | Iacolina et al. 2016 | 5 | 5 | 5 | 3 | 2 |
| Bosnia and Herzegovina | Bih | new | 6 | 6 | 6 | 1 | 1 |
| Switzerland | Che | new | 32 | 32 | 15 | 2 | 0 |
| Germany | Deu | Iacolina et al. 2016 + new | 67 | 66 | 49 | 43 | 29 |
| Spain | Esp | Iacolina et al. 2016 + new | 51 | 49 | 43 | 40 | 24 |
| Estonia | Est | new | 10 | 10 | 10 | 10 | 9 |
| Finland | Fin | Iacolina et al. 2016 | 5 | 5 | 5 | 3 | 2 |
| France | Fra | Iacolina et al. 2016 + new | 41 | 41 | 25 | 19 | 12 |
| Greece | Grc | Iacolina et al. 2016 | 73 | 73 | 48 | 31 | 19 |
| Croatia | Hrv | Iacolina et al. 2016 + new | 29 | 29 | 24 | 24 | 16 |
| Hungary | Hun | new | 3 | 3 | 3 | 3 | 3 |
| Italy | Ita | Iacolina et al. 2016 + new | 17 | 17 | 6 | 4 | 3 |
| Luxemburg | Lux | Iacolina et al. 2016 | 4 | 4 | 4 | 4 | 3 |
| The Netherlands | Nld | Iacolina et al. 2016 | 26 | 25 | 14 | 6 | 0 |
| Poland | Pol | Iacolina et al. 2016 + new | 17 | 14 | 14 | 14 | 13 |
| Portugal | Prt | Iacolina et al. 2016 + new | 23 | 23 | 13 | 13 | 8 |
| Romania | Rou | new | 19 | 19 | 12 | 11 | 10 |
| Russia | Rus | Iacolina et al. 2016 + new | 9 | 9 | 9 | 9 | 9 |
| Serbia | Srb | Iacolina et al. 2016 + new | 8 | 8 | 7 | 6 | 5 |
| Slovakia | Svk | new | 7 | 7 | 5 | 4 | 4 |
| Slovenia | Svn | Iacolina et al. 2016 | 16 | 16 | 10 | 10 | 8 |
| Ukraine | Ukr | new | 1 | 1 | 1 | 1 | 1 |
| **Total** |  |  | **471** | **464** | **330** | **263** | **182** |

B)

| **Population** | **Number of samples** | **Country (abbreviation)** | **Cluster** |
| --- | --- | --- | --- |
| China Northeast | 5 | Chn | Far East |
| Japan | 12 | Jpn | Far East |
| Korea | 5 | Kor | Far East |
| East Russia | 11 | Rus | Far East |
| Samos Island | 11 | Grc | Near East |
| Israel | 4 | Isr | Near East |

C)

| **Pig Breed** | **Number of samples** | **Breed type** |
| --- | --- | --- |
| Calabrese | 10 | Local Italian |
| Casertana | 6 | Local Italian |
| Cinta_senese | 7 | Local Italian |
| Manchado | 7 | Local Spanish |
| Negro_iberico | 14 | Local Spanish |
| Retinto | 10 | Local Spanish |
| Angler_sattle | 9 | standardized |
| Landrace | 30 | standardized |
| Large_white | 27 | standardized |
| Pietrain | 20 | standardized |
