# Supplementary material for: Spatial genetic structure of European wild boar, with inferences on late-Pleistocene and Holocene demographic history
Source: Heredity (Edinb). 2023 Jan 13;130(3):135–44. doi: 10.1038/s41437-022-00587-1 (PMC9981775; doi:10.1038/s41437-022-00587-1)
Supplement: Supplementary file 2 — Supplementary figures [file 41437_2022_587_MOESM2_ESM.docx]

**Spatial genetic structure of European wild boar, with inferences on late-Pleistocene and Holocene demographic history**

Joost F. de Jong^1^, Laura Iacolina^2,3*^, Herbert H.T. Prins^4^, Pim van Hooft^1^, Richard P.M.A. Crooijmans^5^, Sip E. van Wieren^1^, Joaquin Vicente Baños^6^, Eric Baubet^7^, Seán Cahill^8^, Eduardo Ferreira^9^, Carlos Fonseca^9^, Peter M. Glazov^10^, Ida Jelenko Turinek^11^, András Náhlik^12,13^, Victor M. Lizana Martín^14,15^, Boštjan Pokorny^16,17^, Tomasz Podgórski^18,19^, Nikica Šprem^20^, Rauno Veeroja^21^, Ronald C. Ydenberg^22^ and Hendrik-Jan Megens^5^

**Fig. S1** **Compatibility of the two different beadchip versions used in this study to genotype wild boar samples.** The figure shows the first two axis of a PCoA of Hamming genetic distances of European wild boar genotyped with overlapping SNPs of the PorcineSNP60 beadchip version 1 (blue circles) and version 2 (red triangles). If a relevant discrepancy existed between those versions, for example due to mismatches between differently named or mapped SNPs, PCoA would have discriminated beadchip v1 samples from beadchip v2 samples. Instead, samples from the two different beadchips clustered together. Note that samples at the bottom left corner come from an area from which samples were genotyped with beadchip v1 only.


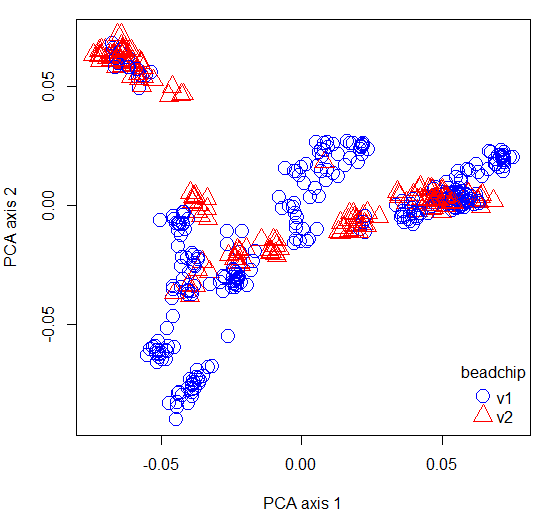


**Fig. S2** **PCoA projection scores, per subset.** The scores are determined by projecting wild boar on the PCoA axis of simulated wild boar and pigs (modern breeds). On the axis of European wild boar and modern breeds, wild boar from most southern regions are projected towards pigs (panel A). Only if wild boar are simulated using allele frequencies specific for each region, southern individuals are projected opposite to pigs (panels B-D). The final PCoA projection score of an individual, used to estimate F_hybrid_ (Fig. 1A) equals the minimum ordination score.
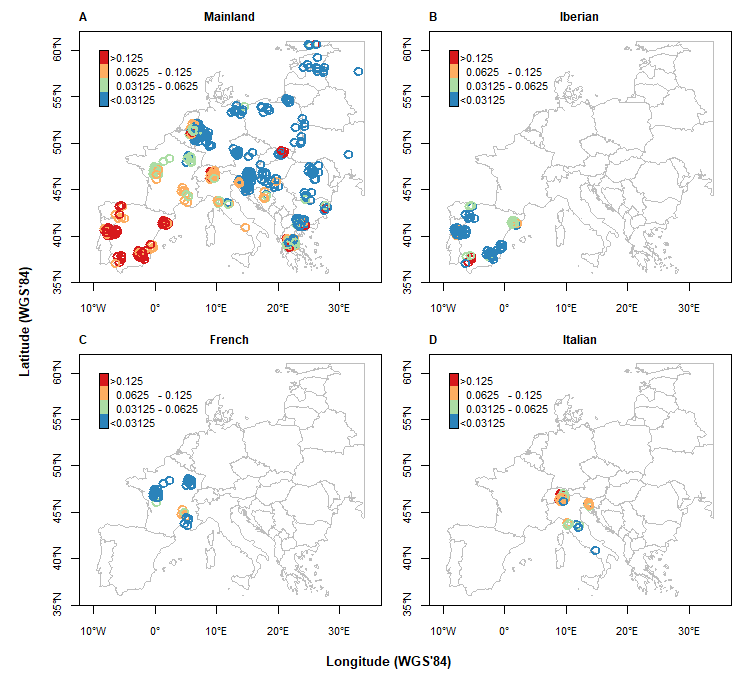


**Fig. S3** Overview of distribution of putative SNP alleles across the wild boar genome, for individuals with different degrees of domestic pig introgression. The F_hybrid_ scores are based on the PCoA projection method (see Fig. 1A). Stripes indicate the presence of one or two putative pig alleles. Putative pig alleles are defined as alleles uncommon in wild boar (MAF < 0.025) but common in pigs (MAF > 0.225). The lower two figures show the Gaussian Kernel densities of SNPs along the chromosome (R base function *density*). Densities are shown for all available SNPs (grey line) and for the SNPs with putative domestic pig alleles.


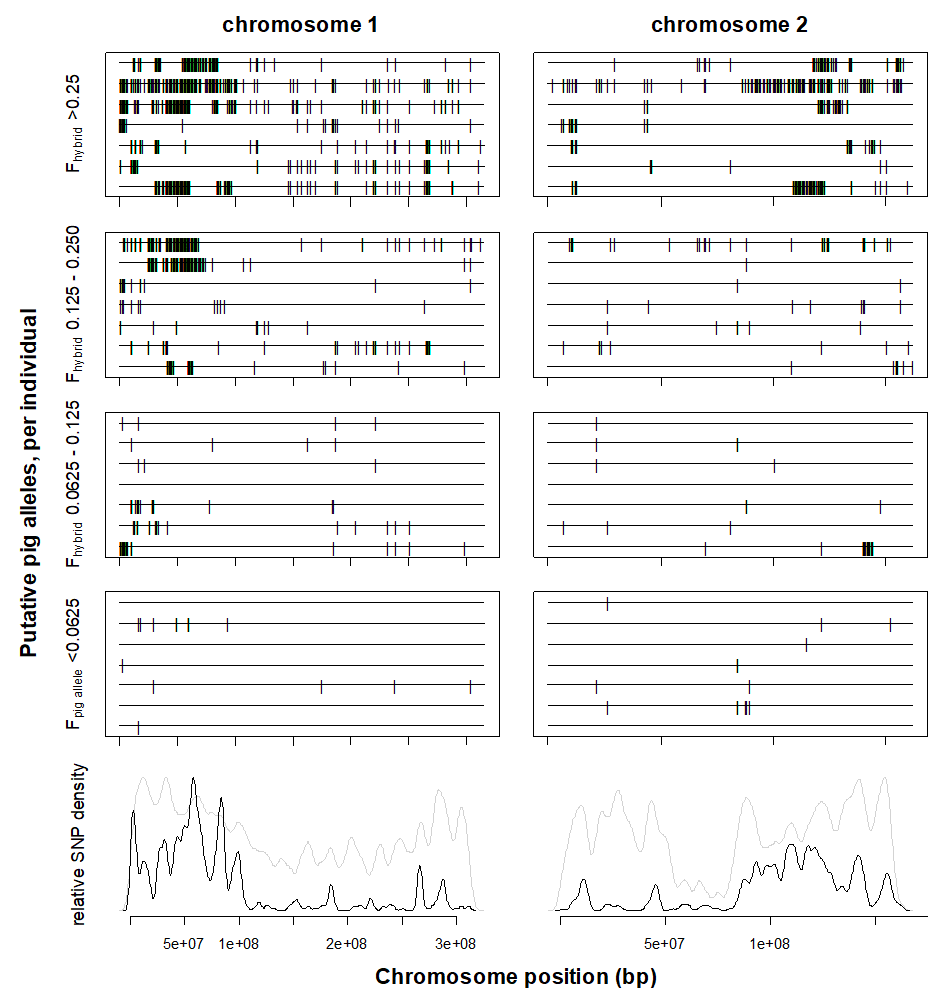


**Fig. S4 Genomic distribution of regions scanned for Runs Of Homozygosity (ROHs).** Grey stripes are the available SNPs. Curves are the Gaussian Kernel density estimates (R base function *density*) of SNP density (calculated per chromosome). Black stripes are regions meeting the following criteria: length > 5 Mb, n SNPs >= 50, mean SNP density > 1 / 100 Kb, inter-SNP distance: < 500 Kb.


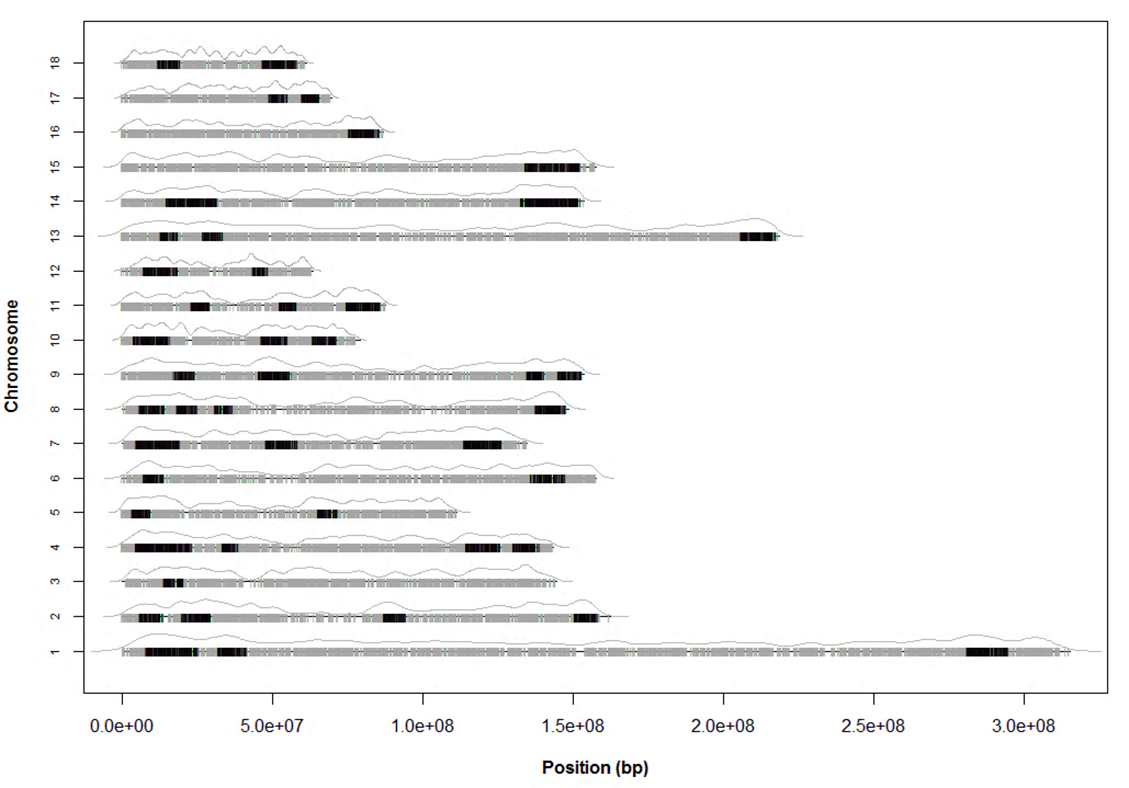


**Fig. S5 PCoA for different inclusion criteria.** (A) All samples, (B) without Iberian wild boar, (C) without hybrid and inbred wild boar, (D) without Iberian, hybrid and inbred wild boar.
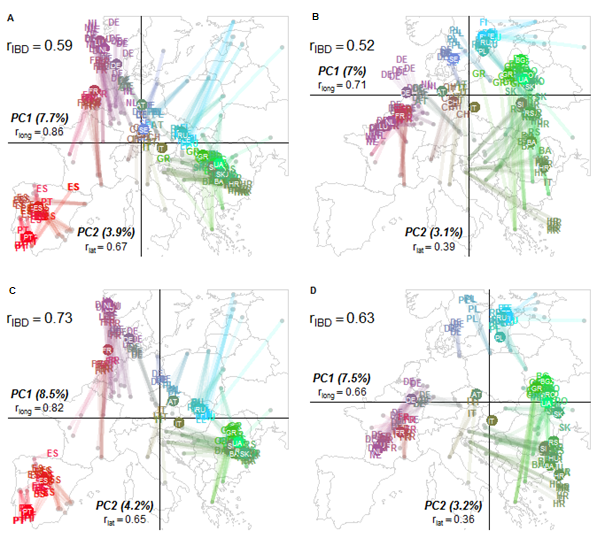


**Fig. S6 Spatial distribution of the samples used for the interpolation of mean allele frequency across Europe (Fig. 4).** Hybrid (F_hybrid_ > 0.062) and inbred individuals (F_ROH_ > 0.062) are excluded.


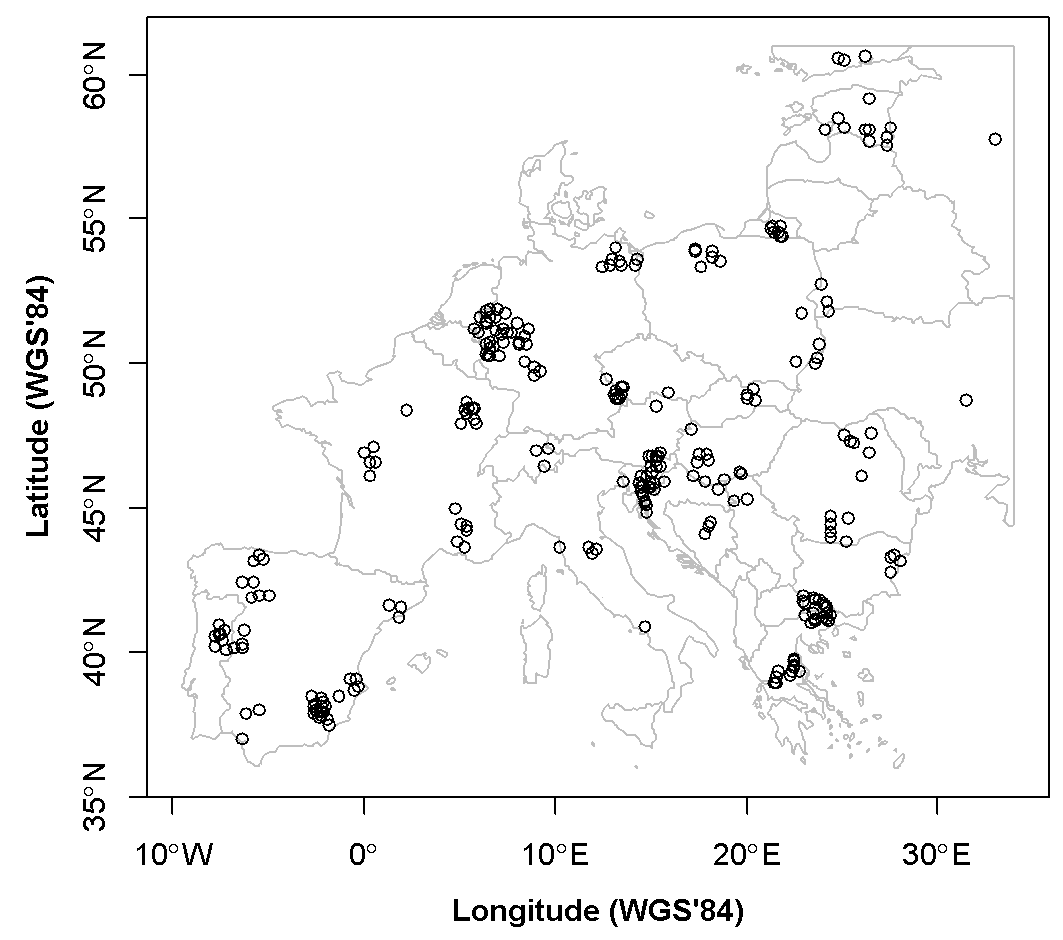


**Fig. S7** **Genetic clusters of wild boar in Europe, overlaid on the forest map of Europe in 1850.** ADMIXTURE analysis of European wild boar, shown for the most likely number of K=5. Symbol size is proportional to sample size. With higher K clusters, Italian wild boar are assigned to separate cluster. Hybrid and inbred wild boar were excluded from the analysis. The underlying map represents the distribution of forests in Europe in 1850 (grey), based on the data modelled by Kaplan et al. (2009).


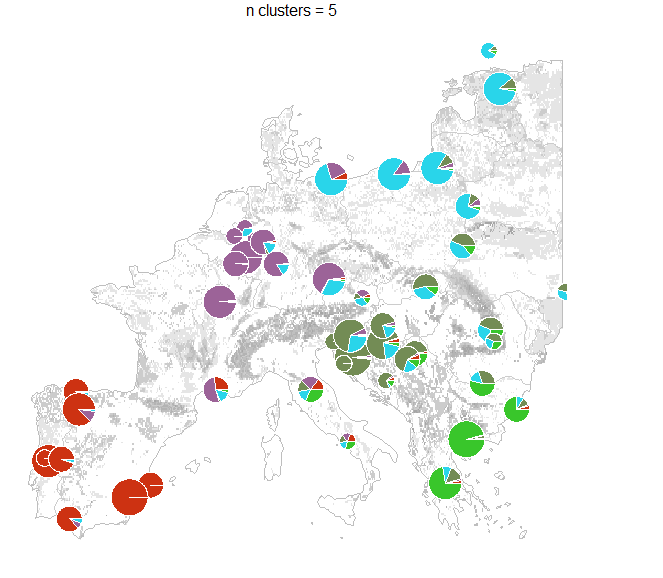


**Fig. S8 (Re)colonization conceptual map.** Maps representing the source populations for the different steps of the (re)colonization of Europe at different time points. Arrows represent the colonization route, circles and ovals the source/remnant populations and dashed lines represent barriers to dispersal. Underlying base map from: <https://commons.wikimedia.org/wiki/File:Western_Europe_DEMIS_topographic_map.svg>

**
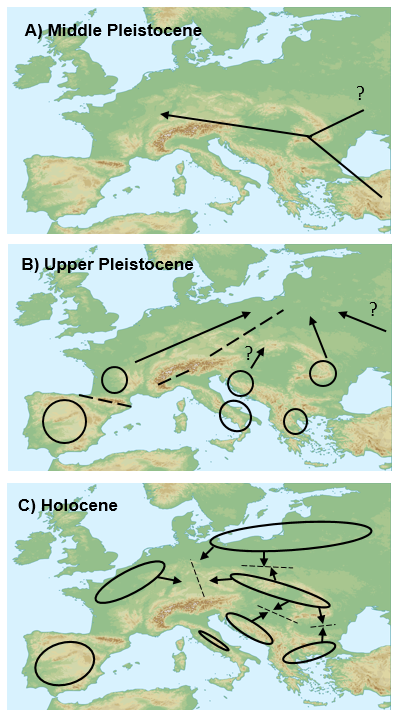
**

**References:**

Iacolina L, Scandura M, Goedbloed DJ, Alexandri P, Crooijmans RPMA, Larson G, et al. (2016) Genomic diversity and differentiation of a managed island wild boar population. Heredity 116: 60–67. <https://doi.org/10.1038/hdy.2015.70>

Keplan JO, Krumhardt KM, Zimmermann N (2009) The prehistoric and preindustrial deforestation of Europe. Quat Sci Rev 28: 3016-3034. <https://doi.org/10.1016/j.quascirev.2009.09.028>
